# Supplementary material for: Complexome profiling of the Chlamydomonas psb28 mutant reveals TEF5 as an early PSII assembly factor
Source: Plant Cell. 2025 Mar 17;37(6):koaf055. doi: 10.1093/plcell/koaf055 (PMC12164590; doi:10.1093/plcell/koaf055)
Supplement: koaf055_Supplementary_Data [file koaf055_supplementary_data.zip › psb28_interactive.zip!/plots/intensity/qualityPlots/peptides/NUOB18_Cre06.g278188.t1.1.html]

### Cre06.g278188.t1.1

Trivial name: NUOB18  
SeqCoverage [median]: 37.20  
Mass: 10.28 kDa  
  
 

| Sequence | MissedCleavage | Mass [Da] | wtIntensity | muIntensity | Score |
| --- | --- | --- | --- | --- | --- |
| ATQEQMDAADVPYHYR | 0 | 1893.83 | 1077.55 | 1235.68 | 117.00 |
| LTSGFSWR | 0 | 952.48 | 263.38 | 1670.09 | 65.04 |
| VAIAIEEK | 0 | 871.50 | 0.00 | 2259.94 | 69.20 |
|  |  |  | 1340.9 | 5165.7 |  |
